# Comparative Study of the Outcome of Buccal Mucosa Graft Urethroplasty and Preputial Flap Urethroplasty for Anterior Urethral Stricture: A Prospective Randomized Study

**DOI:** 10.7759/cureus.55732

**Published:** 2024-03-07

**Authors:** Nandesh Kumar, Ahsan Ahmad, Rohit Upadhyay, Rajesh Kumar Tiwari, Khalid Mehmood, Nikhil Ranjan

**Affiliations:** 1 Department of Urology, Indira Gandhi Institute of Medical Sciences, Patna, IND

**Keywords:** bmg urethroplasty, sexual function, recurrence, infection, idiopathic

## Abstract

Background: Urethroplasty using a buccal mucosa graft (BMG) and a preputial skin flap (PSF) are two common techniques used for the treatment of anterior urethral stricture. The present study compared the efficacy of these two techniques on the basis of success rate, preservation of sexual function, and complications.

Materials and methods: This prospective, randomized, interventional study was conducted on adult male patients diagnosed with non-obliterative anterior urethral strictures of length >2 cm from August 2021 to December 2022. Pre-operative and post-operative work-up done included assessment of the International Prostate Symptom Score (IPSS), Quality of Life (QOL), International Index of Erectile Function (IIEF) Score, Male Sexual Health Questionnaire for Ejaculatory Dysfunction (MSHQ-EJD), Bother score, Urethral Stricture Surgery-Patient-Related Outcome Measure (USS-PROM), and peak urinary ﬂow rate (Qmax) for each patient. Post-operative values for each score were compared with pre-operative values.

Results: Out of 31 patients, 16 underwent BMG urethroplasty, and 15 underwent PSF urethroplasty. The most common cause of stricture in both groups was idiopathic (35.5% and 53.3%). A statistically significant increase in IIEF score was observed in the BMG group in comparison to the PSF group (P<0.0001). The mean IPPS score in USS-PROM has shown a significant drop in BMG (19.6 vs. 17.3; P = 0.020). Hemoglobin drop was significantly higher in PSF than in BMG (2.6 vs. 1.9; P = 0.011). A higher incidence of surgical site infection was reported in the PSF group than in the BMG group (46.7% vs. 12.5%). The average operative time for surgery was higher in PSF than in BMG (154.8 min vs. 145.0 min), respectively. Each group had one patient with a recurrence.

Conclusion: Both techniques are equally good for urethral reconstruction (UR); however, improvement of sexual function is more in favor of the BMG urethroplasty group.

## Introduction

Urethral stricture occurs when the urethra narrows due to scar tissue, causing problems with urination. It is a common problem in men, affecting approximately 200 to 1,200 out of every 100,000 men [1]. This blockage can significantly reduce the patient's quality of life (QoL), affecting sexual function. In extreme cases, there may be chronic retention and bilateral hydroureteronephrosis, resulting in loss of renal function [2]. It is, therefore, essential that urethral strictures be recognized early and appropriately treated.

In the last two decades, urethral reconstruction (UR) using a buccal mucosa graft (BMG) has become popular amongst urologists [3]. The BMG has unique physical characteristics, such as hairlessness. It is easy to harvest and is associated with early uptake, a good graft survival rate, and compatibility with dampness [4]. Before the application of the BMG graft, local skin flaps with robust vascular pedicles were used to substitute the urethral mucosa. The preputial skin flap (PSF) is better than other types of flaps due to its adaptability [5]. Although previous studies have shown equal results for flaps and grafts, harvesting a flap is technically more challenging, associated with higher morbidity, and less preferred by the patient [6]. Results from numerous studies have highlighted the emerging role of BMG in reconstructing the anterior urethra. Controversy still exists regarding the use of buccal mucosa on the less vascular pendulous urethra [6].

Few studies conducted worldwide have compared the efficacy of BMG and PSF groups [7-9]. However, the comparative outcomes in terms of functional improvement, quality of life, and sexual function have not been studied. Therefore, the purpose of this study was to compare these two substitution urethroplasty techniques in terms of success rate, recurrence, complications, and different aspects of sexual function (desire, erection and ejaculation, and satisfaction) in Indian patients.

## Materials and methods

Study design

This prospective, randomized, interventional study was conducted at the Department of Urology, Indira Gandhi Institute of Medical Sciences, Patna, between August 2021 and December 2022. The study was approved by the institutional ethics committee of the Indira Gandhi Institute of Medical Sciences and was performed in accordance with the Declaration of Helsinki and the International Conference on Harmonization guidelines (276/IEC/IGIMS/2021; approved on 05/10/2021). Written informed consent was obtained from all the participants prior to enrollment in this study.

Study participants

Adult male patients were randomized to undergo substitution urethroplasty utilizing a preputial skin flap and buccal mucosa-free graft. Non-obliterative anterior urethral strictures >2 cm of inflammatory, iatrogenic, and idiopathic causes were included in this study. Patients with a diagnosis of balanitis xerotica obliterans, poor penile skin, or any pathology of the oral mucosa with a history of repeated instrumentation or intervention were excluded from the study.

Data collection

Patients who met the eligibility criteria were randomized to undergo BMG or PSF urethroplasty. Pre-operative history was taken, including the International Prostate Symptom Score (IPSS), the International Index of Erectile Function (IIEF) Score, the Male Sexual Health Questionnaire for Ejaculatory Dysfunction (MSHQ-EJD), the Urethral Stricture Surgery-Patient-Related Outcome Measure (USS-PROM), the Bother score, and the Quality of Life score.

Study treatment procedure

Patients who met the eligibility criteria were randomized to receive either BMG urethroplasty or PSF urethroplasty.

BMG Urethroplasty

**Figure 1:**
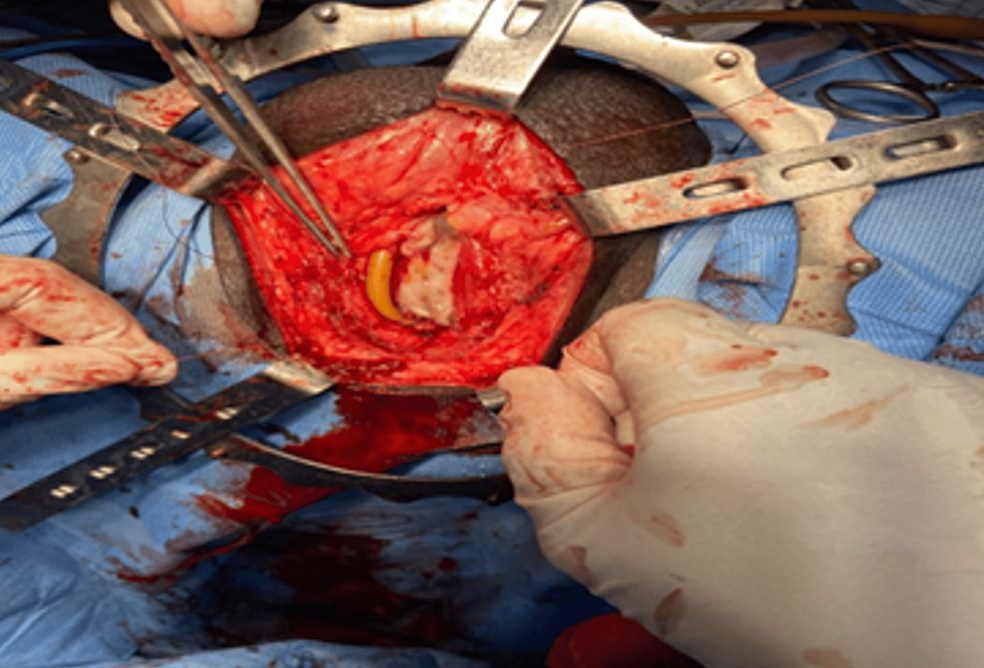
Buccal mucosa graft urethroplasty

Under general anesthesia with nasal intubation and lithotomy position. A pre-operative urethroscopy was done for the evaluation of stricture and to pass the guidewire into the bladder using a pediatric cystoscope. For a better understanding of diseased mucosa, methylene blue was injected into the urethra. A mid-line perineal incision was made, and the bulbospongiosus muscle was separated from the corpus spongiosum. The bulbar urethra, along with the penile urethra, was dissected from the corpora cavernosa, depending upon the length of the stricture. The dorsal urethral surface was exposed by mobilizing and partially rotating the urethra. By proximal and distal extension of urethrotomy, the stricture was opened to its full length until pink mucosa was visible both proximally and distally. On the inner side of the patient's cheek, buccal mucosa was harvested by another surgeon. The graft was sutured to the margins of the opened urethra (Figure 1).

Preputial Flap Urethroplasty

**Figure 2:**
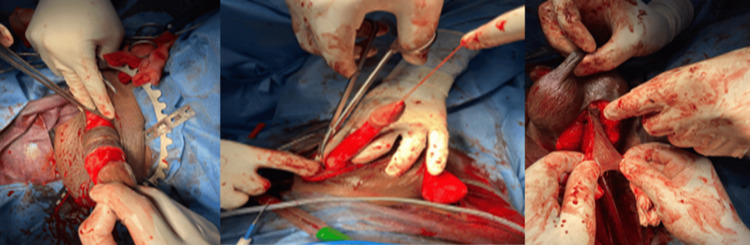
Preputial flap urethroplasty

Under spinal anesthesia in the standard lithotomy position. The diseased part of the urethra was completely mobilized from the corpora cavernosa. The stricture was opened longitudinally at the dorsal aspect. With intraoperative assessment, the length of the stricture was measured. A dartos-based inner preputial flap was developed. After splitting the flap ventrally, it was brought down through a tunnel beneath the penile skin to place it dorsally over the incised urethral segment. Proper mobilization of penile skin allows the flap to reach the most proximal part of the anterior urethra without torsion and without compromising the blood supply. The margins of the flap were sutured to the margins of the urethra. For three weeks, Foley’s catheter and suprapubic catheter were kept indwelling. After the removal of the urethral catheter, SPC was clamped. If the patient voided satisfactorily for one week, then the SPC was removed (Figure 2).

Scores

The IPSS score is based on the answers to seven questions related to urinary symptoms, such as incomplete emptying, frequency, intermittency, urgency, weak stream, strain, nocturia, and one question concerning the quality of life. The answers are assigned points from 0 to 5. The total score can, therefore, range from 0 to 35 (asymptomatic to very symptomatic). The first seven questions of the I-PSS are identical to the questions appearing on the American Urological Association (AUA) symptom index, which categorizes symptoms as: (i) mild (symptom score ≤ 7), (ii) moderate (symptom score range 8-19), and (iii) severe (symptom score range 20-35).

The IIEF is a multi-dimensional self-report instrument widely used to evaluate male sexual function. This standardized and validated 15-item self-evaluation scale provides pre- and post-treatment clinic evaluations of erectile function, orgasmic function, sexual desire, satisfaction in sexual intercourse, and general satisfaction. The male sexual health questionnaire (MSHQ) provides an in-depth assessment of ejaculatory function. The seven-item ejaculatory function domain of the MSHQ is an assessment of the frequency of ejaculation, strength/force of ejaculation, volume of ejaculation, delay of ejaculation, dry ejaculation, pleasure when ejaculating, and pain when ejaculating.

Patient-reported outcome measures are health questionnaires that aim to collect patients' self-assessment of health and of the quality of care delivered before and after the treatment. The USS-PROM questionnaire contains 13 questions in three domains. The first domain includes six questions that are taken from an international consultation on incontinence questionnaire, where each question is scored from 0-4, resulting in a total score between 0 (no symptoms) and 24 (most symptoms) points. This domain also includes one question about dependent quality of life and a visual scale assessment of the urine stream. In the second domain, two questions related to patient satisfaction with the operation’s results are included. The third domain consists of five questions about overall health status and quality of life.

The Clavien-Dindo classification of surgical complications is based on the therapy used to correct a specific complication. It consists of seven grades (I, II, IIIa, IIIb, IVa, IVb, and V).

Further evaluation of patients at three and six months with a similar questionnaire was conducted along with uroflowmetry. No obstructive symptoms on IPSS with a peak urinary flow rate of a minimum of 15 mL/s were reported as a success.

Outcomes

The primary outcome was defined as if the patient was asymptomatic with a maximum urinary flow (*Q*_max_) >15 mL/s. Functional parameters such as IPSS, IIEF Score, MSHQ-EJD, and USS-PROM, oral problems, and complications linked to penile skin were reported as secondary outcomes.

Statistical analysis

Data were analyzed using Statistical Package for the Social Sciences (SPSS) version 16 (IBM Corp., Armonk, NY). Descriptive statistics were used to describe categorical variables (frequency and percentages) and continuous variables (mean and standard deviation [SD]). A qualitative comparison between the groups was done using a paired T-test and a standard T-test. A P<0.05 was considered statistically significant.

## Results

A total of 31 patients were randomly selected to undergo either dorsal BMG urethroplasty (n=16) or PSF urethroplasty (n=15). The mean age was comparable between the two groups (36.3 years and 35.4 years, respectively). The mean BMI was 22.6 kg/m2 and 22.2 kg/m2 in PSF and BMG, respectively. The stricture length between the two groups was similar (5.8 cm in each group). In the PSF group, all strictures were penobulbar, while in the BMG group, two in bulbar and 14 in penobulbar regions were reported. In PSF, the most common cause of urethral stricture was idiopathic (53.3%), followed by post-instrumentation (20.0%) and post-inflammation (26.7%). Idiopathic (37.5%) and post-instrumentation (37.5%) were the most common causes of urethral strictures, followed by post-inflammation (31.3%) in the BMG group. Hypertension was the most common co-morbidity in the study population (38.7%) (Table 1).

**Table 1:** Demographic and baseline characteristics of patients Data presented as mean (SE), unless otherwise specified. BMI: basal metabolic index.

Parameter	Preputial skin flap (n=15)	Buccal mucosa graft (n=16)	P value
Age (years)	36.3 (3.6)	35.4 (2.7)	0.840
BMI (kg/m^2^)	22.6 (0.7)	22.2 (0.8)	0.740
Length of stricture (cm)	5.8 (0.3)	5.8 (0.3)	0.830
Location of stricture, n (%)			
Bulbar	-	2.0 (12.5)	-
Penobulbar	15.0 (100.0)	14.0 (87.5)	-
Etiology, n (%)			
Post-instrumentation	3 (20.0)	6 (37.5)	
Post-inflammation	4 (26.6)	4 (31.2)	
Idiopathic	8 (53.3)	6 (37.5)	
Duration of disease before treatment (months)	33.1 (3.1)	29.6 (3.9)	0.490
Comorbidities, n (%)	[n = 6]	[n = 8]	-
Diabetes mellitus	1.0 (6.6)	1.0 (6.2)	
Hypertension	5.0 (33.3)	7.0 (43.8)	

The mean change in IPSS score in PSF from baseline to six months was 26.2 to 8.2. The IPSS drop in BMG from baseline to six months was 24.5 to 5.9. The quality-of-life score was comparable in both groups. The mean change in the quality-of-life score from baseline to six months was 31.9 in PSF and 41.3 in BMG. The ejaculatory function has shown improvement from baseline in both groups. The change from baseline in the mean IIEF levels in patients from the PSF group was significantly (P<0.0001) higher than in the BMG group. In PSF, the MSHQ EJD improved from a baseline of 10.9 to 17.2 at six months. A similar improvement was seen in BMG, from baseline 10.4 to 18.5 at six months. Bother scores increased in both groups, from baseline 10.9 to 17.2 at 6 months in the first group and 10.4 to 18.5 in BMG, respectively. The USS-PROM shows a higher significant drop in mean IPSS score in BMG than PSF (19.6 vs. 17.3; P = 0.020) (Table 2).

**Table 2:** Pre- and post-operative data Data presented as mean (SE), unless otherwise specified. IIEF: International Index of Erectile Function; IPSS: International Prostate Symptom Score; LUTS: lower urinary tract symptoms; MSHQ-EJD: male sexual health questionnaire for ejaculatory dysfunction; *Q*_max_: peak urinary ﬂow rate; USS-PROM: urethral stricture surgery-patient related outcome measure; VAS: Visual Analogue Score.

Parameter	Preputial skin flap (n=15)	Buccal mucosal graft (n=16)	P-value
IPSS score			
Baseline	26.1 (1.1)	24.5 (1.1)	0.290
3 months	7.5 (1.3)	8.3 (0.7)	
6 months	8.2 (1.0)	5.9 (0.2)	
Mean change from baseline	17.9 (2.7)	18.6 (2.2)	0.436
QOL			
Baseline	5.8 (0.1)	5.4 (0.2)	0.055
6 months	4.6 (0.2)	4.4 (0.5)	
Mean change from baseline	1.2 (0.4)	0.9 (0.4)	0.098
IIEF			
Baseline	20.9 (2.0)	22.8 (2.7)	0.580
6 months	52.9 (1.1)	64.1 (1.3)	
Mean change from baseline	31.9 (4.9)	41.3 (5.2)	<0.0001
MSHQ EJD scale			
Baseline	10.9 (1.2)	10.4 (0.9)	0.193
6 months	17.2 (1.4)	18.5 (1.3)	
Mean change from baseline	6.3 (2.8)	7.7 (2.7)	0.149
Bother score			
Baseline	1.7 (0.1)	1.6 (0.2)	0.475
6 months	4.0 (0.2)	4.5 (0.2)	
Mean change from baseline	2.4 (0.5)	2.8 (0.5)	0.012
USS-PROM Score LUTS			
Baseline	26.1 (0.8)	28.1 (0.8)	< 0.0001
6 months	8.7 (0.8)	8.5 (0.9)	
Mean change from baseline	17.3 (2.6)	19.6 (2.9	0.020
Peeling			
Baseline	3.3 (0.2)	3.8 (0.1)	< 0.0001
6 months	1.6 (0.2)	1.9 (0.2)	
Mean change from baseline	1.7 (0.5)	1.8 (0.4)	0.388
VAS			
Baseline	39.7 (2.5)	36.2 (3.3)	0.002
6 months	75.3 (3.3)	74.4 (3.7)	
Mean change from baseline	35.7 (8.1)	38.2 (10.5)	0.460
*Q*_max_ (mL/s)			
Baseline	5.6 (0.6)	6.5 (0.6)	0.280
6 months	19.5 (1.0)	19.1 (0.7)	
Mean change from baseline	13.9 (2.5)	12.6 (1.7)	0.079
Hemoglobin drop			
Pre-operative	12.7 (0.3)	12.9 (0.3)	-
Post-operative	10.1 (0.2)	11.0 (0.3)	-
Mean change from baseline	2.6 (0.7)	1.9 (0.7)	0.011
Surgical site infections n (%)	7.0 (46.7)	2.0 (12.5)	-
Clavien-Dindo classification of complications n (%)			
Grade 1	13.0 (86.7)	13.0 (81.3)	
Grade 2	2.0 (13.3)	3.0 (18.8)	
Operative time (mins)	154.8 (3.6)	145.0 (4.4)	0.098
Duration of hospital stay (days)	5.5 (0.3)	5.3 (0.2)	0.540

The peeling score was slightly higher in BMG (1.8 vs. 1.6; P = 0.388). The mean drop in VAS was comparable between both groups (35.7 and 38.2, respectively; P = 0.460). The mean difference in Qmax score from baseline to six months was 13.9 mL/s in PSF and 12.6 mL/s in BMG. The hemoglobin drop was significantly higher in the PSF vs. BMG (2.6 vs. 1.9; P = 0.011). A higher incidence of surgical site infection was reported in the PSF group than in the BMG group (46.7% vs. 12.5%). The Clavien-Dindo classification of complications was similar in both groups; 13 patients from each group have reported grade 1 complications. The average operative time for surgery was higher in PSF than in BMG (154.8 min vs. 145.0 min, respectively). The mean duration of hospital stay was comparable between the two groups (5.5 days and 5.3 days; P = 0.540) (Table 2). Overall, the success rate of urethroplasty in this study was 93.54%. The success rate of urethroplasty at six months was comparable between both groups (PSF: 93.33% and BMG: 93.75%). A recurrence was seen in one patient in each group.

## Discussion

In this study, two common urethroplasty techniques, PSF and BMG, were compared. The key observations of the study were: (i) the most common cause of urethral stricture was idiopathic; (ii) the change from baseline in the mean IIEF levels of patients in the BMG group was significantly higher than the PSF group; (iii) there was a consistent decline in the IPPS score in both groups; (iv) the level of hemoglobin drop was significantly higher in the PSF than BMG; (v) a higher incidence of infection at the surgical site was reported in PSF than BMG; and (vi) the average operative time for surgery was higher in PSF than in BMG.

In the present study, the most common cause of urethral stricture was idiopathic, followed by post-instrumentation and post-inflammation. Soliman et al. reported idiopathic stricture as the most common type of urethral stricture in the BMG and PSF groups [9]. A meta-analysis showed idiopathic as the most common cause of urethral stricture, accounting for 51.3% of cases, followed by iatrogenic (32.8%), infective (8.0%), and traumatic (6.6%) [10]. However, a recently published randomized controlled trial by Tyagi et al. reported post-instrumentation as the most common cause, followed by post-inflammation and idiopathic [11].

In this study, an increase in the IIEF score is reported in both groups. In the PSF group, from baseline, 20.9 to 52.9 at 6 months. Similar trends have been reported in the BMG group, as the IIEF score increased from baseline (22.8) to 64.1. A statistically significant increase in IIEF score was observed in the BMG group compared to the PSF group (P<0.0001).

This study reported a consistent decline in the IPPS score in both groups over a three- and six-month follow-up. Symptomatic improvement in terms of decline in IPSS score has been reported in PSF, from baseline 26.1 to 8.2 at six months. Similar trends have been reported in BMG, as IPPS dropped from a baseline of 24.5 to 5.9. A higher IPSS drop is reported in the BMG group, but it is not statistically significant. A prospective randomized study by Soliman et al. reported a similar decrease in the IPSS score, from a mean of 22.8 preoperatively to 4.2 postoperatively in the BMG group and from a mean of 23.3 preoperatively to 5.3 postoperatively in the PSF group [9]. A similar finding was also reported by Hussein et al., wherein there was a significant improvement in the mean IPSS in the BMG group compared to the PSF group (P<0.0005) [12].

Regarding postoperative complications, a higher incidence of surgical site infection was reported in PSF than in BMG in this study. A similar prospective study on 55 patients by Dubey et al. [6] with anterior urethral strictures who had either BMG (n = 27) or PSF (n = 28) urethroplasty noted that two patients in each group experienced postoperative hematoma and one patient in the PSF group had wound infection. In the PSF group, six patients developed superficial penile skin necrosis, while one developed extensive skin loss. In this study, no patients had complete penile skin necrosis. However, five patients had a band of penile skin necrosis at the corona, which healed with conservative management. Studies comparing postoperative complications between PSF and BMG urethroplasty reported a higher incidence of surgical site infections in PSF compared to BMG, which is consistent with the present study findings [13,14].

In the BMG group, a total of 25.7% of patients had minor oral morbidity, out of which four patients developed perioral numbness and one patient developed a mucus retention cyst [6]. However, in this study, only 10% had minor oral morbidity, such as perioral numbness.

Soliman et al. reported that BMG urethroplasty was simpler from a technical point of view. The mean operative time in the BMG group was significantly shorter than in the PSF group (155 min vs. 218.5 min; P<0.001) [9]. Alsagheer et al., evaluating the surgical outcome of substitution urethroplasty using BMG graft and PSF for anterior urethral stricture ≥8 cm, noted that BMG had a shorter operative time than PSF (199.7 min vs. 240.3 min, respectively; P<0.01) [15]. Similarly, in this study, the average operative time was comparatively shorter in the BMG group (145.0 min) than in the PSF group (154.8 min), provided that two surgeons worked simultaneously. Therefore, BMG has a potential advantage in reducing operative time if two surgeons are working simultaneously. In Dubey’s study, the hospital stay was shorter in the BMG group than in the PSF group. Similarly, in this study, the mean hospital stay was shorter in the BMG group than in the PSF group (5.3 vs. 5.5), but with no statistically significant difference (P = 0.540) [6].

Overall, the success rate of urethroplasty in this study was 93.54%. The success rate of urethroplasty is a crucial metric, with studies indicating long-term success rates as high as 85-90% for surgical reconstruction with urethroplasty [16]. The success rate of PSF was 93.33% at six months in our study, as only one patient in PSF had recurrent stricture. Whereas the success rate of BMG urethroplasty was 93.75%, one patient had a recurrence. Predictors of urethral stricture recurrence vary, with incidence rates ranging between 2% and 36.4% and 75% occurring within the first six months post-surgery [17]. A meta-analysis presented by Sharma et al. revealed that BMG was successful in 83.7% of the patients. Data also showed that BMG urethroplasty had significantly higher success rates for strictures in the bulbar region (87.4% vs. 78.0%; P = 0.0001) [18]. Soliman et al. reported an overall success rate of 86.5%. Patients from the BMG group had comparatively higher success rates than those from the PSF group (89.5% vs. 83.3%) [9]. According to Tyagi et al., the success rates of augmentation urethroplasty (AU) with PSG and BMG were comparable (89% vs. 91%; P = 0.70; 95% confidence interval (CI) = 0.33 to 5.21) till the end of the study. Based on the site of the stricture, the success rate was 86% in patients with penile strictures and 72% in patients with bulbo-penile strictures [11].

## Conclusions

On comparing BMG and PSF methods for long-segment anterior urethral stricture reconstructive surgery, both techniques exhibit similar symptomatic improvement, with no statistically significant advantage for either. However, BMG urethroplasty appears to offer better preservation of sexual function. Despite the lack of statistical significance, the preference for BMG suggests potential benefits for long-term outcomes. To draw definitive conclusions, longer-duration follow-ups and randomized studies with adequate sample sizes are recommended. Overall, both BMG and PSF techniques are viable options for long-segment anterior urethral stricture reconstruction.
